# Mechanisms Underlying the Antidepressant Effect of Acupuncture *via* the CaMK Signaling Pathway

**DOI:** 10.3389/fnbeh.2020.563698

**Published:** 2020-12-04

**Authors:** Lu Bai, Di Zhang, Tao-Tao Cui, Ji-Fei Li, Yang-Yang Gao, Nan-Yi Wang, Peng-Li Jia, Hui-yuan Zhang, Zhong-Ren Sun, Wei Zou, Long Wang

**Affiliations:** ^1^The First Clinical Medical College, Heilongjiang University of Chinese Medicine, Harbin, China; ^2^The Second Clinical Medical College, Heilongjiang University of Chinese Medicine, Harbin, China; ^3^Texas Children’s Hospital, Baylor College of Medicine, Houston, TX, United States; ^4^Second Affiliated Hospital, Heilongjiang University of Chinese Medicine, Harbin, China; ^5^First Affiliated Hospital, Heilongjiang University of Chinese Medicine, Harbin, China

**Keywords:** electroacupuncture, depression, CAMK signaling pathway, Baihui, Shenting

## Abstract

The CaMK pathway has been proven to play an important role in regulating cognitive function and emotional response. Acupuncture through the CaMK pathway improves depression-like behavior and the molecular mechanism related to its antidepressant remains to be explored. In this study, we aimed to determine whether the ability of acupuncture at Baihui (GV20) and Shenting (GV24) points to treat depression is related to the regulation of key proteins in the CaMK pathway. A rat model of depression was induced by chronic unpredicted mild stress (CUMS). Model rats in the electroacupuncture group were subjected to acupuncture at the Baihui (GV20) and Shenting (GV24) acupoints once a day for 20 min. Model rats in the fluoxetine group were gavaged with fluoxetine (1.8 mg/kg). Immunohistochemistry and Western blotting assays were used to evaluate immunoreactivity for and the protein expression levels of CaMKII, CaMKIV, and CaM. The results showed that electroacupuncture had a significant effect in rats with depression. Electroacupuncture and fluoxetine regulated the expression of key proteins in the CaMK signaling pathway, which is related to depression, in the hippocampi of rats. This indicates that acupuncture at Baihui (GV20) and Shenting (GV24) may alleviate depressive symptoms and reduce work- and life-related burdens and stress by regulating the CaMK signaling pathway.

## Introduction

Depression, or major depressive disorder (MDD), is characterized by persistent negative thoughts and feelings that disturb mood, cognition, motivation, and behavior. Depression is the leading cause of disability worldwide, affecting over 300 million people (World Health Organization, 2017). According to a report published by the World Health Organization (WHO), MDD is expected to be the leading cause of disability in the world by 2030 (World Health Organization, 2008). According to research, between 1990 and 2013, the number of people suffering from depression and/or anxiety increased by almost 50%, from 416 million to 615 million. Nearly 10% of the world’s population suffers from depression, which accounts for 30% of the world’s nonfatal, fatal disease burden. A new study calculated the cost of treatment and the health of 36 low-, medium-, and high-income people over the 15 years from 2016 to 2030. The results showed that the cost of counseling and antidepressant treatment is estimated to reach $147 billion (Chisholm et al., 2016). The use of currently available antidepressants is limited by the side effects, slow response, and inadequate therapeutic efficacy of these drugs. Antidepressants may not be able to fully alleviate the depressive symptoms or restore the normal functions of patients (Hillhouse and Porter, 2015). Current antidepressants such as fluoxetine may exert their antidepressive effect by increasing neural plasticity (Santarelli et al., 2003; Ampuero et al., 2010). Several mechanisms can explain the therapeutic effects of acupuncture on models of depression. Animal experiments have shown that acupuncture exerts an antidepressant effect by regulating the central nervous activity and 5-HT1A/B receptor expression in various brain regions including the hippocampus, cortex, thalamus, and hypothalamus (Lee et al., 2019). The antidepressant effect of electroacupuncture is accompanied by a significant decrease in the expression of certain NLRP3 inflammasome components and mature IL-1β. Furthermore, electroacupuncture reverses the increase in P2X7R mRNA and protein expression induced by chronic unpredictable mild stress (CUMS) and ameliorates the pathological changes in the hippocampus (Yue et al., 2018). Previous studies have shown that acupuncture, especially electroacupuncture, can effectively treat depression, alleviating depressive symptoms and behaviors to achieve antidepressant efficacy (Sun et al., 2013; Jiang et al., 2017, 2018); however, the mechanism by which electroacupuncture relieves depression is not yet fully understood. On the other hand, a few studies have focused on the CaMK signaling pathway in the central nervous system. A large body of evidence demonstrates that CaMK is involved in learning, memory, and synaptic plasticity (Mayford et al., 1996; Giese et al., 1998) and that synaptic plasticity is central to learning and memory. Recent studies have identified the important roles of the CaMK cascade in neuronal development, plasticity, and behavior (Wayman et al., 2008). Our research has focused on elucidating the antidepressant mechanisms of acupuncture for many years (Long et al., 2019). Our previous evidence-based studies have shown that acupuncture regulates the hypothalamic–pituitary–adrenal axis (HPA axis; Sun et al., 2007), inflammatory cytokines (Di et al., 2017), and neurotrophic factors in the hippocampus to exert an antidepressant effect. The hippocampus has an inhibitory effect on the activity of the HPA axis, and through wider connections with other marginal and frontal regions, it also participates more widely in cognitive and emotional processes. Relevant systematic reviews and clinical studies have shown that electroacupuncture is more efficacious than antidepressants. There is no difference in the curative effect between acupuncture and Western medicine, and acupuncture is associated with few side effects and is well-tolerated. It can also lead to benign changes in hippocampal neuron morphology (Long and Qingbin, 2008; Long et al., 2008). However, the mechanism underlying the antidepressant effects of electroacupuncture has not been confirmed. Therefore, in this study, we established a rat model of depression using a CUMS protocol to evaluate the effects of electroacupuncture on the expression of key factors in the hippocampal CaMK pathway. Our aim was to elucidate the mechanism underlying the antidepressant effect of electroacupuncture and to explore whether electroacupuncture exerts antidepressant effects through the CaMK signaling pathway to provide a theoretical basis for treating depression with acupuncture.

## Materials and Methods

### Animals

Rats were obtained from Heilongjiang University of Chinese Medicine, Heilongjiang, China [Certificate No. SYXK (Hei-longjiang) 2013-012]. Briefly, healthy adult male Sprague–Dawley (SD) rats weighing 180–220 g and aged 6–7 weeks were housed under a 12-h light/dark cycle at 23 ± 2°C and 55 ± 5% relative humidity and provided unlimited access to standard food and water. The rats were housed six per cage in a clean environment. All rats were acclimated to the environment for 1 week before the experiment.

### Experimental Grouping

A total of 120 healthy male SD rats were randomly divided into four groups. The 30 rats in group A (the control group) were randomly divided into the 7-day, 14-day, and 21-day subgroups, with 10 rats in each group. The 30 rats in group B (the CUMS group), the 30 rats in group C (the CUMS + electroacupuncture group), and 30 rats in group D (the CUMS + fluoxetine group) were divided into the same subgroups as in the control group and subjected to the CUMS protocol.

### Induction of Depression by CUMS

A depression model was induced according to the method of Willner et al. (1987) and Hennessy et al. (2001). All model animals were orphaned. The rats were exposed to different stimuli, including limited access to food (for 24 h), persistent water deprivation (for 24 h), tail clipping (180 s, 1 cm from the tail tip), swimming in cold water (10°C, for 5 min), day and night reversal (for 24 h), electric foot shock (voltage 30 V, 5-s shocks with an interval of 5 s for a total of 120 s), and heat (40°C, 5 min) for 21 days, with an average of three exposures to each stimulus.

### Experimental Intervention

**Figure 1:**
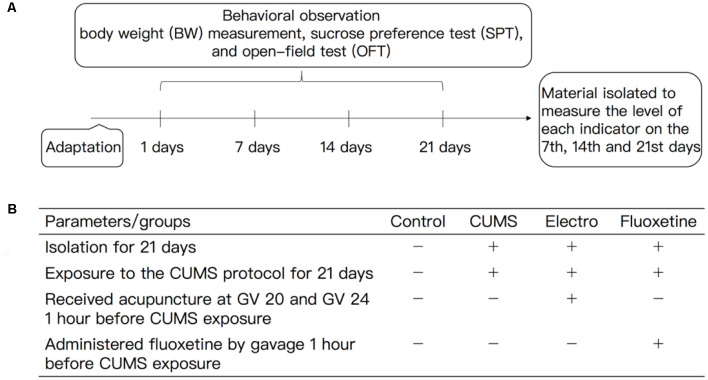
**(A)** Experimental procedures and **(B)** experimental animal intervention method. All the experimental rats were fed for 1 week. A CUMS protocol was used to induce depression in the rats in the CUMS group, electroacupuncture group and fluoxetine group, and all of the rats were raised in isolation except those in the control group. The CUMS rats were exposed to different stimuli during the experimental period. The stimuli mainly included fasting (for 24 h), water deprivation (for 24 h), tail clipping (for 3 min), swimming in cold water (10°C, for 5 min), horizontal turbulence (for 2 min), day and night reversal (for 24 h) and heat exposure (40°C, for 5 min), and the stimuli were presented in an unpredictable manner. The model rats were subjected to the OFT and SPT. The BWs of the rats and other behavioral indexes data were measured at the appropriate time points. The animals in the CUMS group were exposure to the CUMS protocol, during which they were subjected to different stimuli. The animals in the electroacupuncture group were subjected to electroacupuncture for 20 min once a day before stress. The Baihui (GV20) and Shenting (GV24) acupoints, which correspond to human anatomical regions, were located according to “Acupuncture and Moxibustion.” The intensity of acupuncture was sufficient to induce microvibration of the rat head. The animals in the fluoxetine group were administered fluoxetine (1.8 mg/kg, 0.18 mg/ml distilled water) once a day 1 h before the stress exposure. BW, body weight; SPT, sucrose preference test; OFT, open-field test, CUMS, chronic unpredicted mild stress.

The rats in group A (the control group) were not exposed to any stimuli and were provided free access to food and drinking water. Depression was induced in the rats in group B (the CUMS group) as described above (induction of depression by CUMS). The rats in group C (the electroacupuncture group) were subjected to electroacupuncture once a day for 20 min 1 h before daily stress exposure for 21 days. The Baihui (GV20) and Shenting (GV24) acupoints, which correspond to the human anatomical regions, were located according to “Experimental Acupuncture Science” (Guo and Fang, 2016), and 0.25 × 25 mm Hwato disposable acupuncture needles were used. The G6085-II electroacupuncture instrument was used to perform acupuncture at Baihui and Shenting at a depth of 0.5–1 cm. Baihui (GV20) was the positive pole, and the negative pole was Shenting (GV24). The frequency was 2 Hz, and the current intensity was 0.6 mA. The acupuncture strength was sufficient to induce microvibration of the rat head. The rats in group D (the fluoxetine group) were intragastrically administered fluoxetine (1.8 mg/kg, 0.18 mg/ml in distilled water) once a day 1 h before stress exposure for 21 days (Figure 1).

### Reagents and Antibodies

The following materials were used: Hwato acupuncture needles (0.25 mm × 25 mm), the G6085-II electroacupuncture instrument (Qingdao Xinsheng Industrial Company Limited), an electric thermostat (Shanghai Yiheng), fluoxetine (Lilly Suzhou Pharmaceutical Company Limited, batch number: 4501A), a CaMK antibody (Abcam, batch number: AB68234, 100 mg), a calcium/calmodulin-dependent protein kinase II (CaMKII) antibody (CST, batch number 4436S, 100 μl), a calcium/calmodulin-dependent protein kinase IV (CaMKIV) antibody (CST, batch number 4032S, 100 μl), an HRP-conjugated goat anti-rabbit IgG secondary antibody (Shanghai Shengbo Biomedical Technology Company Limited, 100 μl), PBS (Shanghai Shengbo Biomedical Technology Company Limited, 500 ml), normal goat serum (Shanghai Shengbo Biomedical Technology Company Limited, 500 ml), citric acid tissue antigen repair solution (Shanghai Shengbo Biomedical Technology Company Limited, 100 ml), DAB color developer (Beijing Zhongzhuang Jinqiao Biotechnology Company Limited), and the JEM-2100F, transmission electron microscope (Japanese Electronics).

### Behavioral Observation

All behavioral observations were performed in a quiet and dark environment. All behavioral tests were completed by the same inspector who was blinded to the group assignments. Body weight (BW) measurements, the sucrose preference test (SPT), and the open-field test (OFT) were performed at least 12 h after stress exposure at the end of the experimental period.

### BW

Changes in weight relative to baseline were calculated to assess food preference and nutritional status. BW was detected on day 0 before intervention and 7, 14, and 21 days after the intervention throughout the experimental period.

### SPT

The SPT was used to evaluate the anhedonia of the rats. All rats were deprived of food and water for 24 h. Then, they were provided free access to two preweighed bottles, one of which contained 150 ml of sucrose solution (1% w/v) and the other contained 150 ml of pure water, for 24 h. The position of the two solution bottles was changed every 12 h to reduce the effect of b side preference. At the end of the investigation, the bottles containing 1% sucrose solution and pure water were weighted, and the weights were recorded. The SPT was conducted on day 0 before intervention and 7, 14, and 21 days after the intervention. Reduced sucrose consumption was indicative of anhedonia.

### OFT

We evaluated the autonomous locomotor activity of the animals with the OFT to improve the credibility of the behavioral test results. The locomotor activity of each rat was tested in an illuminated open-field apparatus. The open-field apparatus was an 80 × 80 × 40 cm square arena with four black walls and a black base that was divided into 16 × 16 cm equal squares with clear white lines. Each rat was gently placed in the center of the open-field apparatus and allowed to move independently and explore freely for 5 min. The number of squares entered (the numbers of horizontal lines crossed, with three paws in the same square) and the number of times the rat stood (including erect rearing) were monitored and recorded as indicators of locomotor activity and exploratory behavior. The total number of points for each rat related to crossing the apparatus (instances in which three or four paws entered the same square were scored as 1 point) and standing (instances in which the front paws of the rat left the ground at the same time were scored as 1 point) was calculated. After each test, 75% ethyl alcohol was used to clean the open-field apparatus to eliminate the effect of odor cues. The rats were subjected to the OFT on day 0 before intervention and 7, 14, and 21 days after intervention.

### Sample Collection

After the behavioral test was completed, the rats in each experimental group was anesthetized by intraperitoneal injection of 10% chloral hydrate (0.3 ml/100 g, intraperitoneally) on day 7, 14, or 21. After anesthesia, the rats were quickly decapitated. The brain tissues of interest were quickly removed and placed on ice, and the hippocampal tissues from the left and right cerebral hemispheres were separated, quickly sealed in a cryotube, and snap-frozen in liquid nitrogen.

### Immunohistochemistry

The rats were anesthetized by intraperitoneal injection of 10% chloral hydrate (3 ml/kg). After anesthesia, brain tissues were removed, and the hippocampal tissues from the left and right cerebral hemispheres were collected. Dewaxing was carried out after fixation, embedding, slicing, and baking. The sections were placed in a 3% fresh H_2_O_2_ solution at 25°C for 10 min to block endogenous peroxidase activity and then washed three times in 0.01 M PBS for 3 min each. Subsequently, the normal goat serum working solution was applied to the sections, at room temperature for 20 min. The sections were incubated with primary antibody overnight at 4°C and washed three times for 3 min each with 0.01 M PBS. The sections were then incubated with biotinylated secondary antibodies at 37°C for 30 min, washed three times with 0.01 M PBS for 3 min each and developed with DAB developer. The sections were counterstained with hematoxylin, sealed with a neutral gum, and then observed under an optical microscope.

### Western Blot Analysis

The rats were anesthetized with 10% chloral hydrate (3 ml/kg) and decapitated, Hippocampal tissue were removed immediately and placed in 50 μl protein lysis buffer for 20 min until the buffer fully infiltrated the cells. Then, the samples were centrifuged at 4°C at 12,000 rotations/min, and the supernatant was collected. A 2 μl aliquot of the supernatant was used to measure the protein concentration. A BCA protein concentration determination kit was used to determine the protein concentration. First, a protein standard with a concentration of 0.5 mg/ml was prepared and reserved for later use. Then, reagent A and reagent B were mixed at a ratio of 50:1 to prepare a working solution, and the protein samples and the standard were added to separate wells of a 96-well plate. A total of 200 μl of prepared BCA working solution was added and allowed to completely penetrate the protein samples and standards, and the integrated optical density (IOD) value of each well was measured. A 10% mini separation gel was made but mixing 2.7 ml of separation gel buffer and separation gel solution with 60 μl of modified ammonium persulfate solution. After mixing, the gel was poured between two glass plates. Another concentrated gel was made by mixing 0.75 ml of concentrated gel buffer and concentrated gel solution with 15 μl of modified ammonium persulfate solution. After mixing, the gel was poured between two glass plates, and then electrophoresis was performed. The gel was rinsed gently with ddH_2_O and placed together with the glass plate in an electrophoresis tank. Electrophoresis solution (100 ml 10× Tris–glycine + 5 ml 20% SDS + ddH_2_O to 1,000 ml) was added to the tank, the sample wells were rinsed, a pipette was used to draw each sample, the pipette was inserted into each well, and the samples were slowly added to the wells. The pipette was cleaned between each sample to avoid cross-contamination. Furthermore, the same amount of protein was added to each well. The initial voltage was kept at 80 V. When the protein reached the separation gel, the voltage was increased to 120 V. When the protein reached the bottom of the gel, electrophoresis was stopped. Subsequently, a PVGF membrane was soaked in methanol for approximately 10 s, transfer solution (100 ml 10× Tris–glycine + 200 ml methanol + ddH_2_O to 1,000 ml) was prepared, the gel was cut and placed in the tank, ice cubes and transfer solution were added to the tank, and the proteins were transferred for 90 min at 300 mA. After transfer, the membrane was blocked with 5% skim milk at room temperature for 1 h, washed with TBST (50 ml 20 × TBS + 1 ml 1,000 × Tween-20 + ddH_2_O to 1,000 ml) three times for 5 min each, and incubated overnight with primary antibody with shaking at 4°C. The membrane was washed three times with TBST for 5 min each, incubated with secondary antibody for 1 h at room temperature, washed three times with TBST for 15 min each, and exposed to light.

### Data Analysis

All statistical tests were performed using SPSS 17.0 software (IBM, Armonk, New York, NY, USA). All data are expressed as the mean ± SD. Analysis of variance was used for comparisons between multiple groups, and Student’s *t*-test analysis method was used for comparison between two groups. For data that did not conform to a normal distribution or homogeneity of variance, the nonparametric rank sum test was used. For the chi-square test,* P* < 0.05 was regarded as statistically significant, and *P* < 0.01 was regarded as very statistically significant.

## Results

### Behavioral Observations

#### Changes in BW

**Figure 2:**
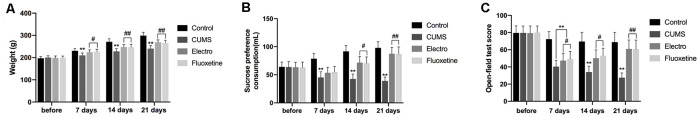
A total of 120 healthy SD rats were selected; 30 were used as control rats, and the remaining 90 were divided into the CUMS group (*n* = 30), electroacupuncture group (*n* = 30), and fluoxetine group (*n* = 30). These 90 rats were used for the construction of a depression model (CUMS) and subjected to the corresponding interventions. Compared with those of the rats in the control group, the OFT scores, sucrose water consumption, and BW of the rats in the CUMS group were significantly lower, indicating that significant depression-like behavior. In the CUMS group, the electroacupuncture group and the fluoxetine group, OFT scores, sucrose water consumption and BW were higher than those in the control group, and there was no significant difference between the groups. The data are expressed as the mean ± standard error of the mean (SD). **(A)** Changes in Body Weight. **(B)** Changes in Sucrose Preference. **(C)** Changes in OFT Scores. **P < 0.01 vs. the control group; ^#^*P* < 0.05, ^##^*P* < 0.01 vs. the CUMS group.

Before the experimental procedures, there were no significant differences in BW among groups. However, there were significant differences in the changes in BW among groups after CUMS exposure. The BW of the rats in the CUMS group was significantly lower than that of the rats in the control group, fluoxetine group, and electroacupuncture group 7, 14, and 21 days after CUMS (*P* < 0.01; *P* < 0.01; *P* < 0.01). Unlike those in the CUMS group, the rats in the electroacupuncture group and fluoxetine group showed weight gain 7, 14, and 21 days after CUMS (*P* < 0.05, *P* < 0.05; *P* < 0.01, *P* < 0.01; *P* < 0.01, *P* < 0.01). There were no significant differences in BW gain between the fluoxetine and electroacupuncture groups (Figure 2A).

#### SPT

There was no significant difference in sucrose preference scores between groups before CUMS model establishment (*P* > 0.05). On day 7, the sucrose preference of the CUMS group was significantly lower than that of the control group (*P* < 0.01). The difference in sucrose preference between the electroacupuncture group and the fluoxetine group was not statistically significant (*P* > 0.05). On days 14 and 21 of treatment, the sucrose water consumption of the CUMS group was significantly lower than that of the control group (*P* < 0.01; *P* < 0.01). Compared with that of the CUMS group, the sucrose water consumption of the electroacupuncture group and the fluoxetine group was significantly higher (*P* < 0.05, *P* < 0.05; *P* < 0.01, *P* < 0.01). The difference in sucrose water consumption between the electroacupuncture group and the fluoxetine group was not statistically significant (*P* > 0.05; Figure 2B).

#### OFT

There was a significant difference in locomotor activity scores in the OFT (combined analysis of horizontal and vertical motion scores) between the CUMS group and the control group, especially on days 7, 14, and 21 after CUMS (*P* < 0.01; *P* < 0.01; *P* < 0.01). It is worth noting that on the 7th day, the scores of rats in the fluoxetine group and the electroacupuncture group were also significantly lower than those in the control group (*P* < 0.01; *P* < 0.01). After 14 days and 21 days of intervention, the spontaneous activity scores of the rats in the fluoxetine and electroacupuncture groups were significantly higher than those of the CUMS group (*P* < 0.05, *P* < 0.05; *P* < 0.01, *P* < 0.01; Figure 2C).

### The Analysis of the Antidepressant Effects of Acupuncture *via* the CaMK Signaling Pathway

**Figure 3:**
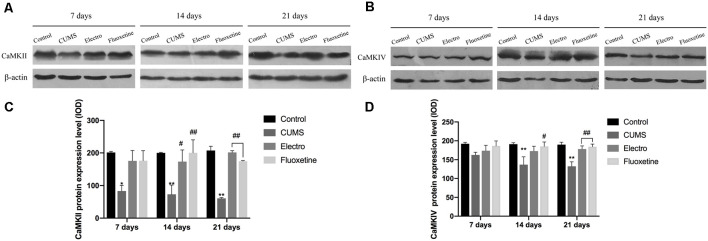
The tissues of interese were removed, and hippocampus tissues from both cerebral hemispheres were collected. Western blotting was used to compare the expression of CaMKII and CaMKIV at each time point on the basis of integrated optical density (IOD) values. On day 7, the expression of CaMKII in the hippocampus in the CUMS group was downregulated compared with that in the control group. However, there was no significant statistical difference in the expression of CaMKIV between the four groups on day 7. On the 14th and 21st days, the expression of CaMKII and CaMKIV in the hippocampus of in the CUMS group was significantly downregulated compared with that in the control group. Compared with that in the CUMS group, the expression of CaMKII in the electroacupuncture group and the fluoxetine group was significantly upregulated, whereas the expression of CaMKIV in the fluoxetine group was also significantly upregulated on day 14. Compared with that in the CUMS group, the expression of CaMKII and CaMKIV in the electroacupuncture group and the fluoxetine group was significantly upregulated on day 21. There were no significant differences in CaMKII and CaMKIV expression between the electroacupuncture group and the fluoxetine group. The data are expressed as the mean ± SD. CaMKII, calcium/calmodulin-dependent protein kinase II; CaMKIV, calcium/calmodulin-dependent protein kinase IV. **(A)** Expression of CaMKII on the hippocampus was shown by western blot. **(B)** Expression of CaMKIV on the hippocampus was shown by western blot. **(C)** CaMKII protein expression level. **(D)** CaMKIV protein expression level. *P < 0.05 vs. the control group; **P < 0.01 vs. the control group; ^#^*P* < 0.05, ^##^*P* < 0.01 vs. the CUMS group.

We evaluated the expression levels of CaMKII and CaMKIV by Western blotting. The Western blotting results showed that the expression of CaMKII in the hippocampus was downregulated in the CUMS group compared with the control group on day 7 (*P* < 0.05). On the 14th and 21st days, the expression of hippocampal CaMKII was significantly upregulated in the electroacupuncture group and the fluoxetine group compared with the CUMS group (*P* < 0.01, *P* < 0.05; *P* < 0.01, *P* < 0.01). CaMKIV expression was also increased in the electroacupuncture and fluoxetine groups (*P* > 0.05, *P* < 0.05; *P* < 0.01, *P* < 0.01). There was no significant difference in CaMKII and CaMKIV expression between the electroacupuncture group and the fluoxetine group (Figure 3).

### Expression Level of Calmodulin 1 (CaM1) in the Hippocampus

**Figure 4:**
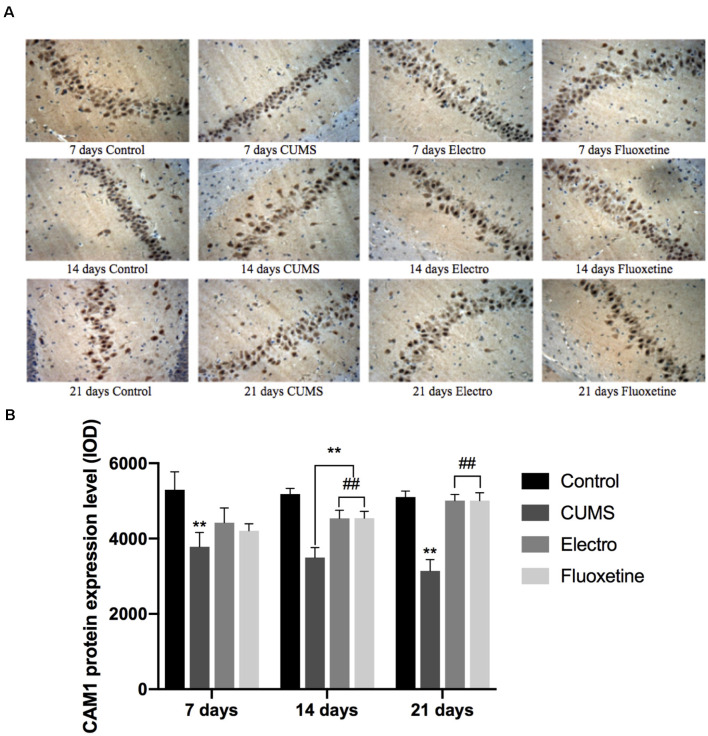
On the 7th, 14th and 21st days after the intervention, the expression in the hippocampus of rats was analyzed by immunohistochemistry. The expression of CaM1 in the CUMS group was significantly lower than that in the control group on day 7, day 14 and day 21. On day 14 and day 21, the CaM1 content in the hippocampus in the electroacupuncture group and the fluoxetine group was significantly higher than that in the CUMS group, and the difference between the electroacupuncture and fluoxetine groups was not statistically significant. The data are expressed as the mean ± SD. **(A)** Expression of CaM1 on the hippocampus was shown by immunohistochemistry. **(B)** CaM1 protein expression level . **P < 0.01 vs. the control group; ^##^*P* < 0.01 vs. the CUMS group.

CaM1 expression in the CUMS group was significantly lower than that in the control group on the 7th, 14th, and 21st days (*P* < 0.01; *P* < 0.01; *P* < 0.01). On days 14 and 21, the CaM1 content in the hippocampus in the electroacupuncture group and the fluoxetine group was significantly higher than that in CUMS group (*P* < 0.01; *P* < 0.01), and there was no significant difference between the fluoxetine and electroacupuncture groups (Figure 4).

## Discussion

Depression is a chronic condition: half of all depressed people have recurrent episodes, and the frequency of episodes and severity of the illness will increase over time (Akil et al., 2018). These recurrent episodes can last from 2 weeks to several years (Kessler et al., 2003; Hasin et al., 2005; Eaton et al., 2008). Unlike most diseases (diabetes, cancer, chronic obstructive pulmonary disease, etc.), depression is not diagnosed based on objective tests (serum chemistry analysis, organ imaging or biopsy), but is diagnosed on the basis of the presence of a highly variable set of symptoms, such as depressed mood, irritability, feeling of inferiority, feelings of hopelessness, and worthlessness, decreased concentration and thinking ability, decreased or increased appetite, weight loss or weight gain, insomnia or hypersomnia, low energy, fatigue, increased agitation, decreased interest in pleasurable stimuli (e.g., sex, food, or social interactions), and recurrent thoughts of death and suicide (Nestler et al., 2002). We carried out animal model experiments based on clinical trials, and the experiments proved that a depression model was successfully constructed. We established a rat model of CUMS-induced depression and assessed related behaviors, including the sensitivity to reward and pleasure and anhedonia with the SPT and BW measurements. Due to the high comorbidity of depression and anxiety (Li et al., 2018), we further tested the ability of the depression model rats to adapt to a new environment in the OFT. Our results indicated that compared to control rats, rats exposed to CUMS exhibited significant loss of appetite, appreciably slower weight gain, poor adaptation to new environments, disinterest in rewarding stimuli, stagnation, and changing in moods as well as susceptibility to low emotions throughout the CUMS protocol. The results of this study are consistent with previous findings suggesting that CUMS may induce depression-like behaviors and effectively mimic depressive symptoms in patients (Luo et al., 2017; Qiao et al., 2017). In this study, although electroacupuncture and fluoxetine were observed to effectively improve the behavior of CUMS rats, a certain period of time was required for the induction of depression by CUMS. Therefore, in future studies, interventions should be applied after successful model establishment.

On the basis of the clinical manifestations of depression, our model was successful. According to relevant research, CaMK is closely related to depression. Multifunctional CaMKs, such as CaMKII and CaM-K cascade members (CaMKK, CaMKI, and CaMKIV), are present in most mammalian tissues but are highly abundant in the brain, where they phosphorylate and regulate a variety of protein substrates (Coultrap and Bayer, 2012). CaMKs are activated *via* the binding of Ca^2+^/CaM (Hell, 2014). Ca^2+^/CaM is essential for studying the etiology and mechanism underlying the effects of antidepressants on depression (Stanley, 2000). CaM is a universally expressed 17-kDa dumbbell-shaped protein (Chin and Means, 2000). The binding of Ca^2+^ to CaM produces a conformational change in CaM that exposes hydrophobic residues, allowing the Ca^2+^/CaM complex to interact with many target proteins and modulate their function. CaMKII has been intensively investigated and is thought to regulate numerous neuronal functions; the effects of CaMKII have been extensively reviewed (Patki et al., 2013; Liu et al., 2014; Huang et al., 2017; Zalcman et al., 2018). Importantly, changes in the expression levels of key proteins in the CaMK signaling pathway play important roles in depression, and the expression levels of CaMKII and CaMKIV proteins in the hippocampus are decreased. In the present study, while we found that electroacupuncture treatment upregulated the protein expression of CaMKII and CaMKIV and alleviated depressive symptoms in CUMS rats, we did not evaluate the expression of the phosphorylated forms of CaMKII and CaMKIV. Electroacupuncture also increased the expression of CaM1. These findings indicate that these proteins may mediate the development of depression to some extent. The differences between the electroacupuncture group and the fluoxetine group were not significant, indicating that both treatment had a similar effect. Other studies have shown that major CaMKIIα phosphorylation sites in TARPγ-8 are disrupted in CaMKIIα-deficient mice, suggesting that an NMDAR activation/CaMKIIα activation/TARPγ-8 phosphorylation cascade is essential for normal plasticity, such as long-term potentiation (LTP), which is considered to be the cellular basis for learning and memory (Park et al., 2016). Thus, a rational direction for future experiments points toward exploring phosphorylation sites and activation pathways of CaMKs and developing a sensitive method for measuring the resulting synaptic changes.

Electroacupuncture has been broadly used to treat various mood disorders for decades (Guo et al., 2016; Kim et al., 2017; Zeng et al., 2018). In this study, we stimulated the Baihui (GV20) and Shenting (GV24) acupoints with electroacupuncture instruments. The Baihui and Shenting acupoints belong to the Du Meridian, and there is a close relationship between Du Meridian and the brain (Zhou et al., 2013; Lu et al., 2016). From the perspective of meridians, the Du Meridian is the sea of the Yangmai, which regulates the pathology of each meridian. It regulates the balance of yin and yang in the body through the brain and has a good effect on the treatment of mental illness. The Baihui acupoint is located on the top of the head, where there is an abundance of neurovascular structures (such as the occipital nerve and frontal branch), and its deep part corresponds to the brain (the motor area of the central cortex and the lateral central lobules; Shen et al., 2011). The Baihui acupoint contains the Baimai party. It can cohere through the acupoints of the body and pass through the yin and yang veins to regulate brain function. The Shenting acupoint is located on the forehead, the vestibule of the mind, and the deep part corresponds to the frontal lobe. The Shenting acupoint is where the Governor’s pulse is ventilated. Acupuncture at the Baihui and Shenting acupoints can affect the mind, relieve depression, and calm the nerves (Lu et al., 2016). Due to the location and function of the Baihui and Shenting acupoints, they are a commonly used acupuncture point for the clinical treatment of depression. Therefore, we used the Baihui and Shenting acupoints for our electroacupuncture intervention in this study. Our study showed that acupuncture had a substantial antidepressant effect, improving depression-related behavior. We also found no significant difference between the electroacupuncture group and the fluoxetine group, indicating that the electrotherapy has a similar effect to the fluoxetine. Studies have shown that fluoxetine is an antidepressant that can induce activation syndrome, which may disrupt sleep (Wong et al., 1995; Nonacs and Cohen, 2003; Wichniak et al., 2017). In some patients, fluoxetine can cause anorexia nervosa (Chojnacki et al., 2015). A meta-analysis showed a positive correlation between fluoxetine use in early pregnancy and major cardiovascular diseases, especially cardiovascular malformations, in infants (Gao et al., 2017). Acupuncture is not entirely free of adverse effects. Some studies have suggested that electroacupuncture is also associated with some side effects, such as hematoma (Linde et al., 2006; Wu et al., 2017), headache (Linde et al., 2006), needling pain (Park et al., 2014), and fainting (Zhang et al., 2010); most of these side effects are minor, and they usually disappear after the end of treatment (Yang et al., 2015; Chan et al., 2017). Electroacupuncture treatment is inexpensive and easy to promote. It can reduce the use of antidepressants, reduce the financial burden on families of patients with depression, and save the country many medical resources.

This study shows that electroacupuncture may have an antidepressant effect by regulating key factors in the CaMK pathway. Further research will focus on the central mechanism underlying the antidepressant effect of electroacupuncture and determine whether it is related to epigenetic modification of hippocampal genes to explore new strategies for the comprehensive treatment of depression.

## Data Availability Statement

The original contributions presented in the study are included in the article, further inquiries can be directed to the corresponding author.

## Ethics Statement

The animal study was reviewed and approved by Laboratory Animal Use and Management Committee of Heilongjiang University of Chinese Medicine.

## Author Contributions

LW and WZ designed the study. LB, DZ, and LW conducted most of the experiments, collected the data, and drafted the article. T-TC, P-LJ, J-FL, Y-YG, and N-YW participated in the discussion and helped with the experiment. H-yZ revised the manuscript. All authors have read and approved the final accepted version of the manuscript.

## Conflict of Interest

The authors declare that the research was conducted in the absence of any commercial or financial relationships that could be construed as a potential conflict of interest.
